# Peripheral Nerve Regeneration Following Crush Injury to Rat Peroneal Nerve by Aqueous Extract of Medicinal Mushroom *Hericium erinaceus* (Bull.: Fr) Pers. (Aphyllophoromycetideae)

**DOI:** 10.1093/ecam/neq062

**Published:** 2011-08-11

**Authors:** Kah-Hui Wong, Murali Naidu, Pamela David, Mahmood Ameen Abdulla, Noorlidah Abdullah, Umah Rani Kuppusamy, Vikineswary Sabaratnam

**Affiliations:** ^1^Institute of Biological Sciences, Faculty of Science, University of Malaya, Kuala Lumpur 50603, Malaysia; ^2^Department of Anatomy, Faculty of Medicine, University of Malaya, Kuala Lumpur 50603, Malaysia; ^3^Department of Molecular Medicine, Faculty of Medicine, University of Malaya, Kuala Lumpur, Malaysia

## Abstract

Nerve crush injury is a well-established axonotmetic model in experimental regeneration studies to investigate the impact of various pharmacological treatments. *Hericium erinaceus* is a temperate mushroom but is now being cultivated in tropical Malaysia. In this study, we investigated the activity of aqueous extract of *H. erinaceus* fresh fruiting bodies in promoting functional recovery following an axonotmetic peroneal nerve injury in adult female Sprague-Dawley rats by daily oral administration. The aim was to investigate the possible use of this mushroom in the treatment of injured nerve. Functional recovery was assessed in behavioral experiment by walking track analysis. Peroneal functional index (PFI) was determined before surgery and after surgery as rats showed signs of recovery. Histological examinations were performed on peroneal nerve by immunofluorescence staining and neuromuscular junction by combined silver-cholinesterase stain. Analysis of PFI indicated that return of hind limb function occurred earlier in rats of aqueous extract or mecobalamin (positive control) group compared to negative control group. Regeneration of axons and reinnervation of motor endplates in extensor digitorum longus muscle in rats of aqueous extract or mecobalamin group developed better than in negative control group. These data suggest that daily oral administration of aqueous extract of *H. erinaceus* fresh fruiting bodies could promote the regeneration of injured rat peroneal nerve in the early stage of recovery.

## 1. Introduction

Peripheral nerve problems are common and encompass a large spectrum of traumatic injuries, diseases, tumors and iatrogenic lesions. The incidence of traumatic injuries is estimated as more than 500 000 new patients annually [1]. Injuries to peripheral nerves result in partial or total loss of motor, sensory and autonomic functions in the involved segments of the body. Nerve crush injury is adequate to investigate the intrinsic cellular and molecular events that intervene in peripheral nerve regeneration, and to assess factors, such as drugs that might enhance the speed of regeneration and the effectiveness of reinnervation [1]. It is known that after the injury due to the tissue destruction, free oxygen radicals increase and cause tissue damage [2].

Traditionally, functional nerve defects have been remedied by many methods, including nerve transfer, nerve grafts, artificial nerve conduit bridging and end-to-side neurorrhaphy [3]. However, these methods only provide a regenerative environment for injured nerves. Recovery of function depends on various local and systemic factors. Regeneration of axons from the proximal stump of an injured nerve to the distal nerve stump is one of the most important factors in reinnervation of peripheral tissue. Recent studies have shown that locally applied neurotrophins can enhance survival of damaged neurons and regrowth of lesioned axons in the central and peripheral nervous systems in rats [4]. However, local treatment is not an ideal treatment pattern. On the other hand, the beneficial effect of systemically administered neurotrophins on axonal regeneration is largely limited by enzymatic degradation. In addition, systemically delivered neurotrophins show unexpected side effects such as the toxicity of the circulating protein [5]. Therefore, it is important to explore substances that can produce neurotrophin-like effects on axonal regeneration without toxicity problem.

The medicinal use of mushrooms has a very long tradition in the Asian countries, whereas their use in the Western hemisphere has been slightly increasing only since the last decades. A scientific journal known as *International Journal of Medicinal Mushrooms*, several books and reviews about medicinal mushrooms and biologically active compounds from mushrooms, and international conferences about this topic confirm this trend [6]. In principle, whole mushrooms (mainly fruiting bodies), extracts (from fruiting bodies or mycelium) and isolated compounds are suitable for use. The material could be obtained by collection from the wild, cultivation of mushrooms in farms and harvesting of the fruiting bodies or by cultivation of mycelium in fermenters with liquid or solid substrates. Extracts could be prepared by extraction of mushrooms (dried or fresh) with suitable solvents. Pure compounds could be obtained by isolation from the natural or cultivated material or by chemical synthesis [6].

Medicinal properties of *Hericium erinaceus* (Bull.: Fr) Pers. (*H. erinaceus*), also known as Lion's Mane, Monkey's Head, Hedgehog Fungus, Satyr's Beard, Pom Pom Blanc, Igelstachelbart and Yamabushitake have been well known for hundreds of years in traditional Chinese and Japanese cooking and herbal medicine to treat various human diseases. The fruiting bodies are composed of numerous constituents such as polysaccharides, proteins, lectins, phenols, hericenones, erinacines and terpenoids. Some of the biological activities of these components have also been studied [7].

The most promising activity of *H. erinaceus* is the stimulation of nerve growth factor (NGF) synthesis by hericenones from fruiting bodies and erinacines from mycelium [8]. An exo-polysaccharide derived from *H. erinaceus* promotes neuronal differentiation and survival [9]. Neurotrophic activities derived from dried fruiting bodies of *H. erinaceus* have also been studied in rat hippocampal slice neurons [10, 11]. Extract of *H. erinaceus* promoted normal development of cultivated cerebellar cells and demonstrated a regulatory effect on the process of myelin genesis *in vitro* after myelin damage [12]. The myelin sheath is a structural component of axon, that is, very important for action potential conduction in the nerve. Injury of myelin compact structure leads to an impairment and severe illness of the nerve system. Our previous study had shown that aqueous extract of the mushroom grown in tropical environment could stimulate neurite outgrowth of the cultured cells of the neural hybrid clone NG108-15 [13]. These findings indicate that *H. erinaceus* may have a potential in stimulation of neurons to regrow in the treatment of senility, Alzheimer's disease, repairing neurological trauma from strokes, improve muscle or motor response pathways and cognitive function.

Research on the medicinal value of *H. erinaceus* grown in Malaysia, a tropical country, is minimal and yet to be explored. To our knowledge, no information is available on the nerve regeneration and repair property of the locally grown mushroom *H. erinaceus*. Therefore, the aim of the study is to assess the peroneal nerve regeneration activity of aqueous extract of *H. erinaceus* fresh fruiting bodies in adult female Sprague-Dawley rats after crush injury.

## 2. Methods

### 2.1. Fruiting Bodies of *H. erinaceus* and Preparation of Aqueous Extract

*Hericium erinaceus* fresh fruiting bodies were obtained from a mushroom farm in Tanjung Sepat, Selangor, Malaysia. It was cultivated on the medium containing rubberwood sawdust, rice bran and calcium carbonate in the mass ratio of 400 : 8 : 5. The inoculated bags were placed in a well-ventilated mushroom house at (27 ± 2) to (32 ± 2)°C. About 300 g of fresh fruiting bodies per 800 g of substrate per bag were harvested after 60 days of spawn run (Cheng Poh Guat, personal communication). Fresh fruiting bodies were boiled with distilled water at a ratio of 1 : 1 for 30 min with agitation, left covered for 30 min, cooled and filtered [14].

### 2.2. Principle of Animal Grouping

The use of rats was approved by the Animal Care and Use Committee of Faculty of Medicine, University of Malaya, Approval Number ANA/16/03/2007/MDKN(R). Forty adult female Sprague-Dawley rats weighing 180 ± 20 g were randomly assigned into four groups of 10 rats each. Negative control group received daily oral administration of distilled water (10 mL kg^−1^ body weight per day), experimental group received low or high dose of aqueous extract of fresh fruiting bodies (10 or 20 mL kg^−1^ body weight per day) and positive control group received mecobalamin (130 *μ*g kg^−1^ body weight per day) using a stainless steel feeding needle for 14 days to function as pre-treatment before surgery.

### 2.3. Surgical Procedure

**Figure 1:**
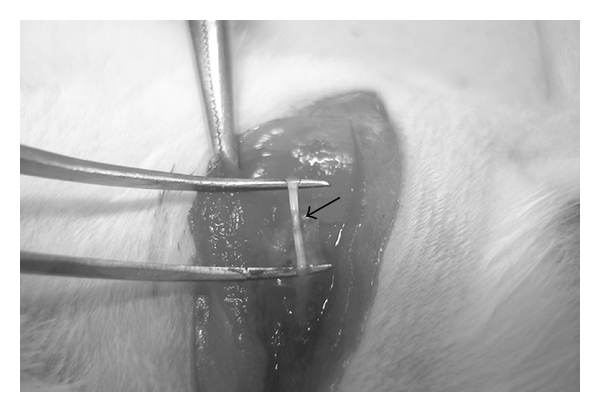
Complete crush of peroneal nerve is confirmed by presence of a translucent band (as indicated by an arrow) across the nerve.

After 14 days of pre-treatment, the rats were anesthetized with an intraperitoneal injection of 3.5% chloral hydrate (10 mL kg^−1^ body weight), then shaved and washed with antiseptic solution before positioning for surgery. The right sciatic nerve and its two major branches were exposed through a gluteal muscle-splitting incision. A crush injury was created using a fine watchmaker forceps no. 4 for 10 s on the peroneal nerve at 10 mm from extensor digitorum longus (EDL) muscle and complete crush was confirmed by presence of a translucent band across the nerve (Figure 1). The incision was then closed in layers (muscle and skin) with absorbable sutures. All operations were performed on right limb and the left limb served as an unoperated control. After closing the incision with sutures, veterinary wound powder (Vet UK Ltd) was applied to wounds. After surgery, distilled water, aqueous extracts or mecobalamin was continuingly fed for another 20 days. All rats were observed for general well being and had *ad libitum* access to food and water throughout the study.

### 2.4. Functional Assessment of Hind Limb Recovery

#### 2.4.1. Walking Track Analysis

**Figure 2:**
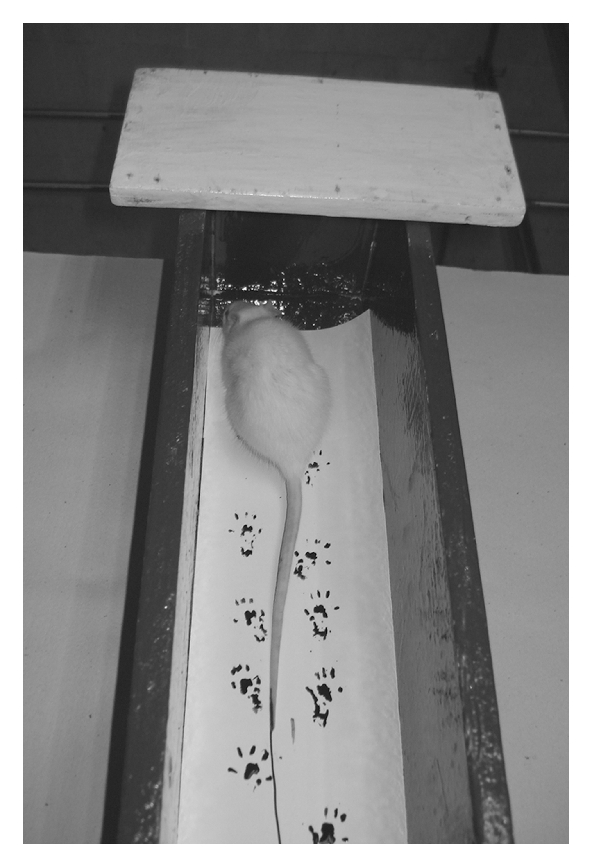
Walking track apparatus. Rat in an 8.2 × 42 cm walking track apparatus lined with white office paper. After the hind limbs of the rat are dipped in Chinese ink, the rat walks towards the darkened end of the corridor.

Rats were allowed conditioning trials in a walking track (8.2 × 42 cm) darkened at one end. White office paper cut to the appropriate dimensions was placed on the bottom of the track. The rat's hind limbs were dipped in Chinese ink, and the rat was permitted to walk down the track, leaving its hind foot prints on the paper (Figure 2). Foot prints were obtained before surgery (day 0) and on day 4, 7, 10 and 14 after surgery as rats showed signs of recovery.

Peroneal functional index (PFI) is based on multiple linear regression analysis of factors derived from measurements of walking tracks in rats with peroneal nerve injury. The factors that contributed to PFI were print length factor (PLF) and toe-spread factor (TSF). Paired measurements of the print length (distance from heel to toe) (PL) and the toe-spread (distance from the first to fifth toes) (TS) were taken for the unoperated/normal (N) foot and the corresponding operated/experimental (E) foot [15]

(1) $$\text{PFI}=174.9\left(\frac{\text{EPL}-\text{NPL}}{\text{NPL}}\right)+80.3\left(\frac{\text{ETS}-\text{NTS}}{\text{NTS}}\right)-13.4$$

A complete recovery of function was determined when the PFI for each group plateaued or returned to its pre-surgery value.

### 2.5. Peroneal Nerve Removal and Immunofluorescence Staining

Qualitative evaluation of peroneal nerve regeneration was achieved by immunofluorescence staining of neurofilaments. Neurofilaments are a type of intermediate filament that serves as major elements of the cytoskeleton supporting the axon cytoplasm. Right peroneal nerves were carefully dissected out and their proximal and distal ends were identified on day 14 after surgery. The contralateral peroneal nerves were also obtained. A series of 20 *μ*m-thick longitudinal sections were cut on a cryostat microtome at –20°C and mounted on poly-l-lysine coated slides. The sections were fixed with freezing acetone for 20 min, then washed three times for 5 min each with 0.01 M phosphate buffer saline containing 0.3% Triton X-100 (washing buffer).

For the assessment of peroneal nerve regeneration, sections were incubated in 10% normal sheep serum in washing buffer (blocking buffer) for 1 h at room temperature. The sections were then incubated with rabbit anti-neurofilament 200 polyclonal antibody (1 : 80 dilution in blocking buffer, Sigma, St. Louis, MO, USA) at 4°C for 20 h in humidity chamber, washed with washing buffer and followed by further reaction with the FITC-conjugated secondary antibody sheep anti-rabbit IgG (1 : 160 in blocking buffer, Sigma, St. Louis, MO, USA) at room temperature for 1 h. After the same washing procedure, sections were coverslipped with antifade reagent. All sections were examined with a Nikon Eclipse 80i microscope under fluorescence illumination using FITC filter and images were analyzed by Nikon's Imaging Software, NIS-Elements.

### 2.6. Microscopic Examination of Neuromuscular Junction

EDL muscles of unoperated and operated limbs were removed on day 14 after surgery. The muscles were fixed by pinning at resting length in buffered-formol calcium for 6 h and immersed in 10% sucrose solution for cryoprotection. Frozen sections (50 *μ*m-thick) were cut longitudinally in a cryostat microtome at –20°C and stained for neuromuscular junction by combined silver-cholinesterase method [16]. Sections were incubated in acetylthiocholine iodide solution in order to stain acetylcholinesterase in the motor endplates. Nerve fibers/axons were stained by impregnation in 10% silver nitrate solution. With this procedure, nerve fibers are stained black, the motor endplates in brown and the muscle fibers in yellow. An endplate was considered to be polyneuronally innervated when two or more axons were approaching from the same or from different directions and could be traced separately to their intramuscular nerve bundle [17]. In each muscle, 100 motor endplates were examined at random whether they were contacted by one or several axon terminals. The number of motor endplates that was polyneuronally innervated was expressed as a percentage of the total number of innervated endplates.

### 2.7. Statistical Analysis

The means of data were subjected to a one way analysis of variance (ANOVA) and the significance of the difference between means was determined by the Duncan's multiple range tests at 95% least significant difference (*P* < .05).

## 3. Results

None of the rats in all groups showed any sign of infection or foot ulceration at any time throughout the experiment. Normal gait was recorded as the hind limb toes fully spread in each group before surgery. Crush injury to the peroneal nerve results in paralysis of the EDL muscle. Flexion contracture (drop foot) was observed due to the lack of dorsal flexion of the ankle. The rats tend to drag the dorsum of their foot until reinnervation of nerve fibers into EDL muscle.

### 3.1. Functional Recovery Enhancement in Treated Groups

**Figure 3:**
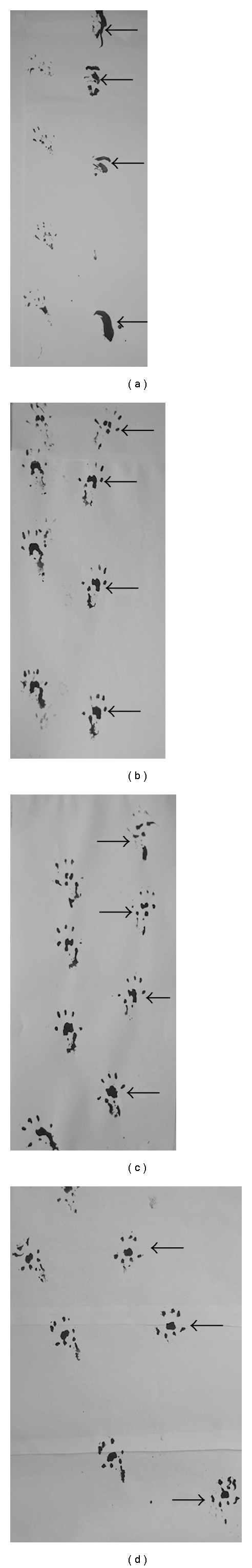
Walking tracks of foot prints after 4 days of right peroneal nerve crush injury. Arrows indicate foot prints of the operated limb. (a) Foot prints in negative control group—distilled water (10 mL kg^−1^ body weight per day). The palsy after interruption of the peroneal nerve is characterized by flexion contracture of the paws (drop foot), absence of toe-spreading reflex and some dragging of the operated limb. (b) Foot prints in low dose of aqueous extract group—*H. erinaceus* fresh fruiting bodies (10 mL kg^−1^ body weight per day). (c) Foot prints in high dose of aqueous extract group—*H. erinaceus* fresh fruiting bodies (20 mL kg^−1^ body weight per day). Toe-spreading and clear foot prints of the operated limb are demonstrated on the walking tracks. (d) Foot prints in positive control group—mecobalamin (130 *μ*g kg^−1^ body weight per day). Clear footprints of the operated limb can be seen.

Functional evaluation showed that recovery in the aqueous extract groups or mecobalamin group began on day 4 while the crushed limb in the negative control group remained dysfunctional. Rats in negative control group showed clumping of toes and dragging of injured foot (Figure 3(a)). These rats were recorded as having unmeasurable walking tracks. On the other hand, aqueous extract groups (Figures 3(b) and 3(c)) or mecobalamin group demonstrated toe-spreading and clear foot prints on the walking tracks (Figure 3(d)).

Analysis of PFI as shown in Table 1 indicated that return of hind limb function occurred by 14 or 17 days after crush injury in five rats each in negative control group. Rats treated with aqueous extract experienced return of function by 10 and 14 days after injury in eight rats and two rats, respectively in low dose group, or six rats and four rats in high dose group. Those treated with mecobalamin also experienced return of function by 10 and 14 days after injury in seven rats and three rats, respectively. When the group's mean PFI were compared at each time interval, the mean PFI of the treated rats was significantly less than the negative control group on day 0, 7, 10 and 14 (*P* < .05). There was no significant difference in PFI between rats in low dose and high dose groups at any time studied (*P* > .05). Print length is shorter at first and will increase back to normal with time or as functional recovery takes place [18].

### 3.2. Promotion of Peroneal Nerve Regeneration in Treated Groups

**Figure 4:**
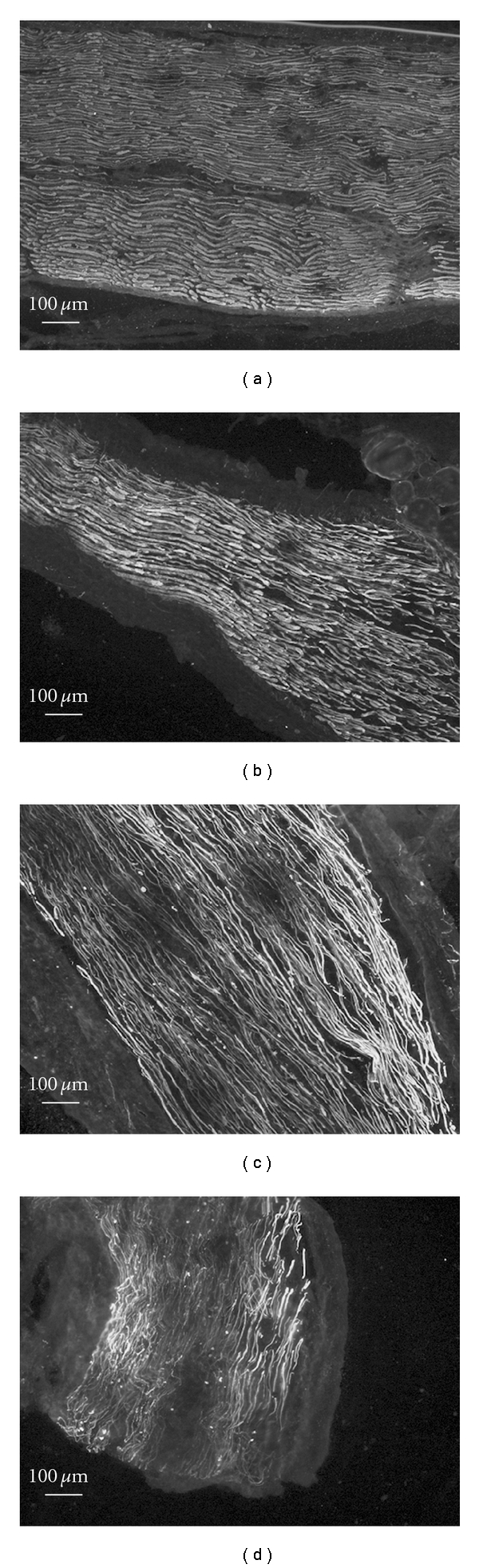
Representative photomicrographs of longitudinally sectioned peroneal nerves distal to the injury site after 14 days of peroneal nerve crush injury and the pathologic scale used for depicting these extents of injury or axon loss. The green fluorescent strands represent individual axon fibers stained with anti-neurofilament 200. 10x magnification. (a) 0 = normal nerve of unoperated limb. (b) 1 = mild axonal damage of operated limb. (c) 2 = moderate axonal damage of operated limb. (d) 3 = severe axonal damage of operated limb.

Anti-neurofilament immunohistochemistry was used to compare the peroneal nerve regeneration in four different groups. Microscopic evaluation of axon was performed in a double-blind fashion by three individuals. Each peroneal nerve was graded for damage on a qualitative 4-point scale [19]. Normal nerve received a score of 0 (Figure 4(a)). Mild, moderate, and severe axonal damage received a score of 1 (Figures 4(b)), 2 (Figure 4(c)) and 3 (Figure 4(d)), respectively. Axons from each group were evaluated to determine the proportion of nerves with an injury greater than or equal to the moderate (≥2) level. In normal peroneal nerve section from unoperated limb, axons appeared to have normal morphology, arranged more densely and uniform neurofilament immunostaining. In crushed peroneal nerve section, axonal regeneration distal to the site of injury can be observed. Regenerating axons sprout aberrantly and form tangled mass or neuromas. Nerve fibers are nonparallel. Two rats in low dose of aqueous extract or mecobalamin group, three rats in high dose of aqueous extract group and eight rats in negative control group demonstrated moderate or severe axonal damage after 14 days of crush injury.

### 3.3. Improved Development of Neuromuscular Junction in Treated Groups

**Figure 5:**
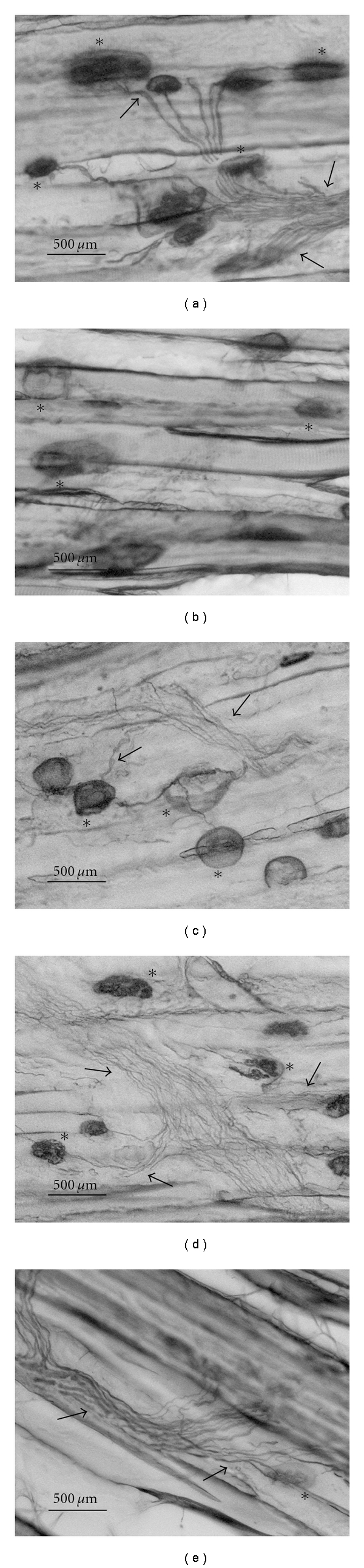
The morphology of silver-cholinesterase stained longitudinal section of extensor digitorum longus (EDL) muscle of rat after 14 days of peroneal nerve crush injury. Arrows indicate the axons. Asterisks indicate the motor endplates. 20x magnification. (a) Normal unoperated limb. Axons bundles are clear and compact. (b) Operated limb in negative control group—distilled water (10 mL kg^−1^ body weight per day). Wallerian degeneration can be detected. (c) Operated limb in low dose of aqueous extract group—*H. erinaceus* fresh fruiting bodies (10 mL kg^−1^ body weight per day). Loose axon bundles indicate regeneration process is on-going. Polyneuronal innervation can be seen. (d) Operated limb in high dose of aqueous extract group—*H. erinaceus* fresh fruiting bodies (20 mL kg^−1^ body weight per day). The presence of motor endplates contacted by either one or more than one axon terminal can be observed. (e) Operated limb in positive control group—mecobalamin (130 *μ*g kg^−1^ body weight per day). Axon bundles are more compact, regeneration process is more advanced compared to aqueous extract group.

The development of muscle reinnervation in the EDL muscle by axons after 14 days of crush injury was assessed by a combined silver-cholinesterase stain. With this method the acetylcholinesterase in the endplate is stained and the axons are impregnated with silver.  Figure 5(a) shows an example of a normal EDL muscle section from unoperated limb. The motor endplates in the EDL muscle are located in the midbelly region of the muscle. The endplates have ellipsoid shapes and the cholinesterase-positive spots are distributed over the entire endplates.

An important difference between muscles of the groups occurs with respect to the regenerating axons. As shown in Figure 5(b), EDL muscles of negative control group contained a mixture of degenerating and regenerating axons, and migration of macrophages to remove degenerated myelin and axon fragments, a process called Wallerian degeneration [1]. Functional connection between motor neuron and EDL muscle fibers has not reestablished at this stage. In rats treated with aqueous extracts, high density of regenerating axons reinnervating motor endplates can be observed (Figures 5(c) and 5(d)). This indicates reestablishment of connection between motor neuron and EDL muscle fibers, leading to functional recovery. In mecobalamin group, axon bundles are more compact and regeneration process is more advanced compared to aqueous extract groups (Figure 5(e)).

In EDL muscle of rats treated with low dose and high dose of aqueous extracts, 16.0 and 19.0% of the muscle fibers were innervated by more than one axon terminal, respectively. On the other hand, EDL muscle of rats treated with mecobalamin had 10.2% of muscle fibers innervated by more than one axon. Higher percentage of polyneuronal innervation of motor endplates was found in the EDL muscle of rats treated with aqueous extracts than those treated with mecobalamin (*P* < .05). However, the phenomenon was not observed in negative control group as Wallerian degeneration was still taking place.

## 4. Discussion

Peripheral nerves may be subjected to crush injuries in a variety of circumstances, including motor vehicle accidents, fractures, dislocations and natural disasters such as earthquakes [20]. After injuries to peripheral nerves, axons and myelin sheaths distal to the lesion are degraded. The degenerative products are eliminated by the cooperative action of denervated Schwann cells and infiltrating macrophages. Wallerian degeneration serves to create a microenvironment favoring axonal regrowth. Schwann cells within the endoneurial tubes of the distal nerve dedifferentiate towards a non-myelinating proliferative phenotype that over-express growth factors, cell adhesion molecules and extracellular matrix to promote regeneration [1].

In contrast to nerve transection injury, nerve crush injury causes less severity, because it leaves the basement membrane of Schwann cells surrounding the original nerve fibers intact and thus, despite the disrupted axon cylinder, enables Schwann cells to provide pathways to guide the regenerating axons [21]. Although crushed peripheral nerves keep anatomical continuity, regenerate spontaneously and somehow reinnervate their target tissues, the longer it takes for the crushed nerve to reinnervate their target tissues, the greater the chance of permanent denervation atrophy of the target tissues. Therefore, accelerated nerve regeneration is crucial to obtain satisfactory functional outcomes [22].

Functional deterioration following crush injury is not only related to the impact of the crush itself, but also includes other important components such as ischemia of the limb. Studies on crush injury models in peripheral nerves have shown better functional recovery when therapies were directed against ischemia-reperfusion injury by using antioxidants, lipid peroxidation inhibitors and anti-inflammatory agents [23]. With this in mind, the present study evaluated the nerve regeneration activity of *H. erinaceus* which possesses antioxidant activity as an alternative herbal pharmacotherapy for peripheral nerve repair [24].

The functional recovery as assessed by PFI scores in couple with histological examination of regenerated nerves and target EDL muscle revealed that aqueous extract promoted peripheral nerve regeneration. It was also noted that the neuroprotective effect of aqueous extract was approaching to those elicited by mecobalamin.

The hind limb function served by the sciatic nerve and its branches in the rat can be quantitatively, reliably and easily assessed by gait analysis through foot prints [18]. The clinically relevant outcome after peripheral nerve injury is the functional recovery of end organ or muscle which is the ultimate test of nerve regeneration. If physical contact is restored between a motor neuron and its muscle, function is usually reestablished [25]. Carlton and Goldberg introduced the PFI [26], which was later modified by Bain et al. [18]. PFI method is suitable for evaluation of a complete lesion of the peroneal nerve which produces a short and narrow foot print due to the lack of dorsal flexion of the ankle and extension of the toes [25]. Although each group demonstrated improvement during the post-surgery period, complete functional recovery was not attained in negative control group until 14 days after injury. Aqueous extract or mecobalamin provides a quicker functional recovery by 4–7 days earlier than negative control group. In spite of this, there was no significant difference in PFI values among all treated groups, showing that different doses of aqueous extract might not have significant effect on functional recovery.

Numerous neurofilament immunopositive axons were seen in peroneal nerve sections from operated limb of aqueous extract and mecobalamin groups. The density of neurofilament immunopositive areas in negative control group was greatly decreased. In fact, nearly all rats in this group sustained at least moderate axonal loss after 14 days of injury.

During the early stages of mammalian ontogeny, muscle fibers are innervated by more than one axon [27]. Polyneuronal innervation is then replaced by mononeuronal innervation in the course of development by mechanical activity of the muscle fiber [28], so that in the rat most muscle fibers are contacted by only one axon by the end of the second week of life [17]. Polyneuronal innervation is rare in normal skeletal muscle. However, it affects a high number of muscle fibers and motor neurons during the first stages after nerve section and regeneration [29]. As the rat recovers its motor function, withdrawal of polyneuron will lead to mononeuronal innervation. If we take the percentage of polyneuronal innervation as a measure of motor endplate maturation, it can be concluded that the rate of maturation after peroneal nerve crush is accelerated in mecobalamin group as compared to aqueous extract groups. Functional connections of severed axons to regenerate and facilitate adequate target reinnervation of EDL muscle was speeded up in treated groups.

Natural products have been traditionally accepted as remedies due to popular belief that they present minor adverse effects [30]. Mushrooms have always been prepared for medicinal use by hot water extraction in traditional Chinese medicine. The extraction was with hot water as in brewing of teas or decoctions. This method is used to prepare extracts of commonly used mushrooms including shiitake, maitake, cordyceps, coriolus and reishi. Hot water extraction has been used for all the well-known products such as lentinan and LEM from shiitake, Maitake d-fraction and MaitakeGold 404 from maitake, and PSP from *Coriolus versicolor* [31].

Aqueous extract is comprised of polysaccharides including acidic heteroglycans, *β*-d-glucans and glucuronoxylomannan. Polysaccharides perform numerous functions in various cell types such as neuritogenesis, peripheral nerve regeneration and muscle reinnervation following a sciatic nerve lesion [32]. Moreover, from a toxicological point of view, water is safer than organic solvents such as acetone, chloroform and methanol.

Sugar and polysaccharide contents in Malaysian grown *H. erinaceus* have been quantified by Choong et al. [33]. The high-performance liquid chromatography (HPLC) analysis of the hot water crude extract showed arabinose as the major component with minor components of glucose, rhamnose, deoxyribose and galactose. The presence of free arabinose in hot water crude extract has not been previously reported. Their finding showed that the fruiting bodies of locally grown *H. erinaceus* contained polysaccharides components which were mainly arabinose and not glucose as mentioned in some studies from China [34].

The dose of 10 mL or 20 mL kg^−1^ body weight per day is based on repeated trials or historical practices. In a study performed in Korea, single- and repeated-dose toxicity studies of Erinacol, the water extract of *H. erinaceus* cultivated with Artemisia iwayomogi have been evaluated according to “Guidelines for Toxicity Tests of Drugs and Related Materials" of Korea Food and Drug Administration using Sprague-Dawley rats [35]. Erinacol up to the limited dose of 5000 mg extract kg^−1^ neither induced death, clinical signs and necropsy findings, nor affected body weight gain and organ weights. The yield of 10 g of fresh fruiting bodies after boiling with 10 mL of distilled water was about 10 mL of aqueous extract. Ten grams of fresh fruiting bodies is equivalent to 1 g of dried powder. Therefore 10 or 20 mL kg^−1^ body weight per day is comparable to 1000 or 2000 mg of dried powder per kg body weight per day. In the present study, the doses of aqueous extract have not shown any signs of immediate danger and exhibited no toxicity in rats.

**Figure 6:**
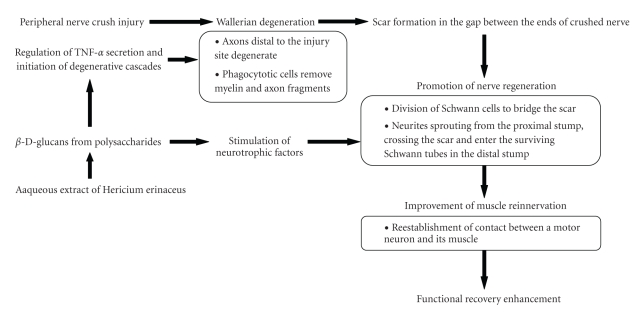
Hypothetical diagram showing the possible effects of aqueous extract of *H. erinaceus* fresh fruiting bodies in promoting peripheral nerve regeneration following crush injury.

With regard to its assumed mechanism of action, the improved regeneration observed after aqueous extract treatment may be related either to a direct neurotrophic factors-like activity or to the promotion of the effects of nerve-derived neurotrophic factor. Neurotrophic factors or regeneration-promoting factors have been suggested to play an essential role in the outcome of degeneration and regeneration processes in the peripheral nervous system, both to ensure proper innervation of the target tissues and to improve remyelination [36]. It may also affect the adherence of platelets and macrophages and the release of cytokines such as tumor necrosis factor (TNF), leading to decreased permeability and tissue edema and better capillary perfusion (Figure 6).

By taking natural products into consideration, the repair effect of the traditional Chinese medicinal herb, *Achyranthes bidentata* Blume root aqueous extract on regeneration of the crushed rabbit common peroneal nerve was studied by Ding et al. [37] and crushed mouse sciatic nerve by Yuan et al. [22] using a combination of electrophysiological assessment and histological investigation. The root extract could accelerate peripheral nerve regeneration in a dose-dependent manner. Lumbricus and Radix Hedysari aqueous extracts have been shown to produce a positive effect on the motor function recovery and conductivity recovery following sciatic nerve clamping injury by increasing the total number of regenerated myelinated nerve fibers in adult rats [38, 39]. Lumbricus is a cold and slightly salty traditional Chinese medicine that derived from the abdomen of earthworm while Radix Hedysari is the dry root of *Hedysarum polybotrys* Hand.-Mazz. Jiang et al. also [40] demonstrated that chitooligosaccharides, the biodegradation product of chitosan, promoted peripheral nerve regeneration with the desired functional recovery in the rat sciatic nerve crush injury model.

From this study, we have shown that aqueous extract of *H. erinaceus* fresh fruiting bodies, administered at a non-toxic dose of 10 or 20 mL kg^−1^ body weight per day increased the rate of recovery after peripheral nerve injury. However, treatment with different doses of aqueous extract did not have a statistical significant difference in recovery as assessed in behavioral experiment and histological examinations. Therefore, low dose of 10 mL kg^−1^ body weight per day would be sufficient in facilitating functional recovery after peripheral nerve injury. Patients who receive *H. erinaceus* may experience a more expeditious improvement in the quality of life and a more complete functional recovery after injury. Moreover, by taking mecobalamin for the treatment of nerve injury gives rise to side effects such as gastrointestinal and dermatological problems [41]. Future research models will focus on direct effect and mechanism of action of *H. erinaceus* on peripheral nerve regeneration and refining strategies to enhance regeneration.

## Funding

University of Malaya for Fundamental Research Grant Scheme FP023/2008C and Postgraduate Research Grant PS150/2008B; Ministry of Science, Technology and Innovation, Malaysia for Science Fund 12-02-03-2050.

## Figures and Tables

**Table 1:** Return of function following crush injury to the peroneal nerve as shown by PFI.

Group	PFI values					
	Day 0	Day 4	Day 7	Day 10	Day 14	Day 17
Negative control	−15.63 ± 4.21^(a)^	Unmeasurable due to dragging of operated foot	−50.36 ± 7.13^(a)^	−32.71 ± 5.27^(a)^	−21.52 ± 8.88^(a)^ [5]	−18.88 ± 6.14 [5]
Aqueous extract	−10.01 ± 3.40^(b)^	−52.88 ± 12.34^(a)^	−21.44 ± 5.56^(b)^	−11.30 ± 4.49^(b)^	−10.71 ± 2.43^(b)^
(low dose)				[8]	[2]
Aqueous extract	−9.28 ± 2.74^(b)^	−48.72 ± 9.72^(a)^	−25.47 ± 6.42^(b)^	−12.46 ± 6.28^(b)^	−10.18 ± 4.11^(b)^
(high dose)				[6]	[4]
Mecobalamin	−10.35 ± 1.80^(b)^	−41.40 ± 5.11^(b)^	−28.27 ± 7.21^(b)^	−20.99 ± 7.05^(c)^	−11.98 ± 1.66^(b)^
				[7]	[3]

PFI of rats treated with aqueous extracts of *H. erinaceus* fresh fruiting bodies or mecobalamin returned to pre-surgery values 4–7 days earlier than negative control. Values on day 0 are before surgery. Data are expressed as means ± standard deviation (*n* = 10 for day 0, 7 and 10 in all groups, day 4 in aqueous extract or mecobalamin group, day 14 in negative control group; *n* = 2, *n* = 4 and *n* = 3 for day 14 in low dose of aqueous extract, high dose of aqueous extract and mecobalamin group, respectively; *n* = 5 for day 17 in negative control group). Brackets indicate number of rats with PFI which had returned to pre-surgery values. Means with different letters in a same column are significantly different (*P* < .05, one-way ANOVA).
