# Corrigendum to “Peripheral Nerve Regeneration Following Crush Injury to Rat Peroneal Nerve by Aqueous Extract of Medicinal Mushroom* Hericium erinaceus* (Bull.: Fr) Pers. (Aphyllophoromycetideae)”

**DOI:** 10.1155/2018/9820769

**Published:** 2018-12-16

**Authors:** Kah-Hui Wong, Murali Naidu, Pamela David, Mahmood Ameen Abdulla, Noorlidah Abdullah, Umah Rani Kuppusamy, Vikineswary Sabaratnam

**Affiliations:** ^1^Institute of Biological Sciences, Faculty of Science, University of Malaya, Kuala Lumpur 50603, Malaysia; ^2^Department of Anatomy, Faculty of Medicine, University of Malaya, Kuala Lumpur 50603, Malaysia; ^3^Department of Molecular Medicine, Faculty of Medicine, University of Malaya, Kuala Lumpur, Malaysia

The article titled “Peripheral Nerve Regeneration Following Crush Injury to Rat Peroneal Nerve by Aqueous Extract of Medicinal Mushroom* Hericium erinaceus* (Bull.: Fr) Pers. (Aphyllophoromycetideae)” [1] was found to present some of the same results, without citation, as another article by the same authors, Kah-Hui Wong et al. [2]. There is an overlap of 2000 words and Figures 1, 2, 3(a), 3(b), and 3(d) in* Evidence-Based Complementary and Alternative Medicine* (2011) are identical to Figures 1, 2, 5Aii, 5Bi, and 5Bii in the* International Journal of Medicinal Mushrooms* (2009).

The authors said that Figures 5Aii, 5Bi, and 5Bii in the* International Journal of Medicinal Mushrooms* article [2] show the outcome of functional recovery to enable standardized collection of data. However, the full experiments on functional recovery were not completed at the time of publication of the* International Journal of Medicinal Mushrooms* article (two experimental groups were reported) and, therefore, these figures were included in the* Evidence-Based Complementary and Alternative Medicine* article [1]. The* Evidence-Based Complementary and Alternative Medicine* article comprises the results of functional recovery with four experimental groups and morphological findings to complement behavioral analysis/functional recovery. The authors added that the images in both articles are their own work and were not modified. They have been used in both articles to emphasize the methodology and to standardize the collection of data in the one continuous experiment they conducted. As it was the authors' own protocol and measurement criteria, they did not cite the first article.

An institutional investigation confirmed these issues. The editorial board agreed to publish a corrigendum.
